# All-Cause and Cardiovascular-Related Mortality in CKD Patients With and Without Heart Failure: A Population-Based Cohort Study in Kaiser Permanente Southern California

**DOI:** 10.1016/j.xkme.2023.100624

**Published:** 2023-03-09

**Authors:** Albert S. Yu, Katherine J. Pak, Hui Zhou, Sally F. Shaw, Jiaxiao Shi, Benjamin I. Broder, John J. Sim

**Affiliations:** 1 Department of Internal Medicine, Kaiser Permanente Los Angeles Medical Center, Los Angeles, CA; 2 Department of Research and Evaluation, Kaiser Permanente Southern California, Pasadena, CA; 3 Department of Clinical and Health Systems Science, Kaiser Permanente Bernard J. Tyson School of Medicine, Pasadena, CA; 4 Department of Quality and Clinical Analysis, Southern California Permanente Medical Group, Pasadena, CA; 5 Division of Nephrology and Hypertension, Kaiser Permanente Los Angeles Medical Center, Los Angeles, CA

**Keywords:** Cardiovascular mortality, chronic kidney disease, heart failure, mortality

## Abstract

**Rationale & Objective:**

Heart failure and chronic kidney disease (CKD) frequently coexist reflective of the strong interplay between these organ systems. A better understanding of the prevalence of different types of heart failure (preserved and reduced ejection fraction) and their subsequent mortality risks among advanced CKD patients would provide important epidemiologic insights and may pave the way for more focused and proactive management strategies.

**Study Design:**

Retrospective cohort study.

**Setting & Population:**

Patients aged ≥18 years with incident CKD (estimated glomerular filtration rate ≤45 mL/min/1.73 m^2^) with and without heart failure in a large integrated health care system in Southern California.

**Exposure:**

Heart failure, heart failure with preserved ejection fraction (HFpEF), heart failure with reduced ejection fraction (HFrEF).

**Outcomes:**

All-cause and cardiovascular-related mortality within one year of CKD identification.

**Analytical Approach:**

HRs were estimated using Cox proportional-hazards model for risk of all-cause mortality and Fine-Gray subdistribution hazard model for risk of cardiovascular-related mortality within 1 year.

**Results:**

The study cohort included 76,688 patients with incident CKD between 2007 and 2017, of which 14,249 (18.6%) had prevalent heart failure. Among these patients, 8,436 (59.2%) had HFpEF and 3,328 (23.3%) had HFrEF. Compared with patients without heart failure, the HR for 1-year all-cause mortality was 1.70 (95% CI, 1.60-1.80) among patients with heart failure. The HRs were 1.59 (95% CI, 1.48-1.70) for patients with HFpEF and 2.43 (95% CI, 2.23-2.65) for patients with HFrEF. Compared with patients without heart failure, the 1-year cardiovascular-related mortality HR for patients with heart failure was 6.69 (95% CI, 5.93-7.54). Cardiovascular-related mortality HR was even higher among those with HFrEF (HR, 11.47; 95% CI, 9.90-13.28).

**Limitations:**

Retrospective design with a short 1-year follow-up period. Additional variables including medication adherence, medication changes, and time-varying variables were not accounted for in this intention-to-treat analysis.

**Conclusions:**

Among patients with incident CKD, heart failure was highly prevalent with HFpEF accounting for over 70% among patients with known ejection fraction. Although the presence of heart failure was associated with higher 1-year all-cause and cardiovascular-related mortality, patients with HFrEF were the most vulnerable.

## Graphical abstract

**Figure:**
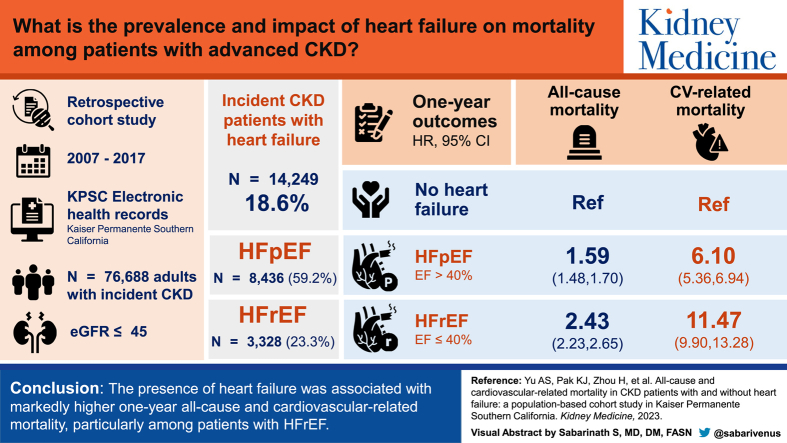

**Plain Language Summary**

Among a large diverse real-world population of patients with advanced chronic kidney disease (CKD) having estimated glomerular filtration rate <45 mL/min/1.73 m^2^, we found that 18.6% had prevalent heart failure where preserved ejection fraction accounted for over 70% among patients with known ejection fraction. Age and sex adjusted 1 year mortality rate was 11 times higher among CKD patients with heart failure than CKD patients without heart failure. All-cause mortality and cardiovascular-related mortality risk was highest among heart failure reduced ejection fraction followed by heart failure preserved ejection fraction. Given the advancements in therapies for heart failure, future studies are needed to assess the efficacy of drugs in real-world populations with CKD. We hope that such future work will provide more insights into the evolving care for heart failure and CKD, ultimately to help better understand and manage this vulnerable population.

Cardiovascular disease is the leading cause of death in patients with chronic kidney disease (CKD).^1^, ^2^, ^3^ An estimated 40%-50% of all deaths in patients with CKD stage 4 and 5 is attributable to cardiovascular-related mortality compared with only 26% in those with normal kidney function.^2^ Furthermore, the rate of hospitalizations among patients with CKD is 60% higher in the presence of cardiovascular disease compared with those without cardiovascular disease.^4^ One of the primary manifestations of cardiovascular disease is heart failure, which has a bidirectional interaction with CKD, termed “cardiorenal syndrome.” Up to 40% of CKD patients who initiate dialysis have heart failure, and among those without heart failure, the annual incidence is estimated at 20%.^4^^,^^5^ A better understanding of the epidemiology of patients with cardiorenal syndrome may improve ways to provide targeted management strategies to this vulnerable population.

Heart failure is a clinical entity that can be categorized as heart failure with preserved ejection fraction (HFpEF), defined as ejection fraction greater than 40%, or heart failure with reduced ejection fraction (HFrEF), defined as ejection fraction less and or equal to 40%.^6^ Multiple cohorts have suggested that HFpEF has been trending higher in the general community and now accounts for up to half of all cases of heart failure,^7^^,^^8^ with differences in prevalence based on age, sex, and comorbid conditions.^4^^,^^9^^,^^10^ A meta-analysis has suggested that the prevalence of HFpEF compared to HFrEF is similar in patients with advanced CKD, accounting for approximately 55%.^11^ The pathophysiologic mechanism of HFpEF is contributed by a proinflammatory state leading to stiffening of the myocardium with increased filling pressures, whereas in HFrEF, there is also decreased myocardial contractility.^12^ In patients with both heart failure and CKD, these mechanisms are further complicated by a complex, bidirectional neurohormonal interaction between the 2 organ systems often referred to as the cardiorenal syndrome.

Using a large diverse population within a real-world environment, we sought to determine the prevalence of heart failure (both HFpEF and HFrEF) in patients with incident advanced CKD. Furthermore, we compared 1-year all-cause and cardiovascular-related mortality within this population.

## Methods

### Study Design

A retrospective cohort study was conducted from January 1, 2007-December 31, 2017 within Kaiser Permanente Southern California (KPSC), which is a large integrated health care system with over 4.8 million members cared for at 14 hospitals and >200 outpatient offices. Information used in this study was collected from the KPSC electronic health records as part of routine clinical care, KPSC administrative files (Table S1). The study was approved by the KPSC institutional review board and exempted from informed consent (IRB # 10591).

### Population

**Figure 1:**
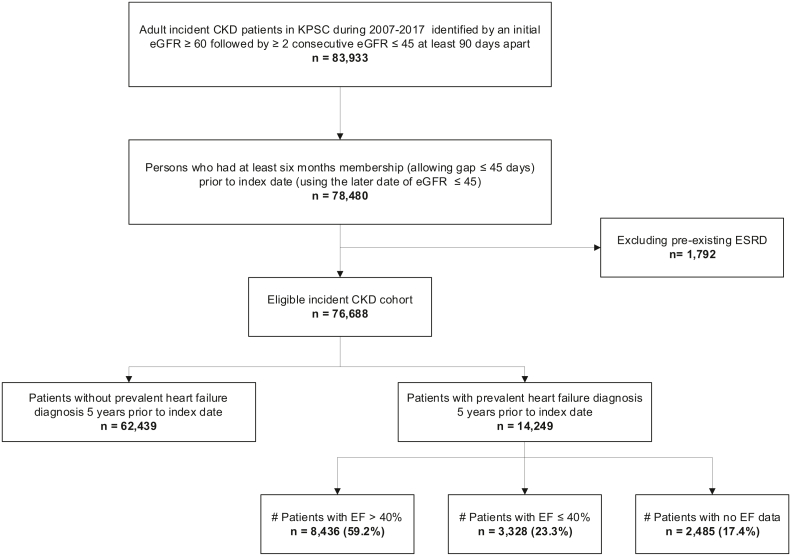
Between 2007-2017, incident CKD patients identified if they had 2 consecutive eGFR measurements ≤45 (mL/min/1.73 m^2^) at least 90 days apart, with a prior eGFR ≥60. A total of 76,688 patients (52.0% non-Hispanic White, 23.4% Hispanic, 13.3% Black, and 8.8% Asian) were identified with incident CKD, of which 14,249 (18.6%) had a diagnosis of heart failure. Among CKD patients with heart failure, 8,436 (59.2%) were HFpEF, whereas 3,328 (23.3%) were HFrEF. Abbreviations: CKD, chronic kidney disease; EF, ejection fraction; eGFR, estimated glomerular filtration rate; ESRD, end-stage renal disease; HFpEF, heart failure with preserved ejection fraction; HFrEF, heart failure with reduced ejection fraction.

Patients (aged ≥18 years) were identified as having incident CKD if they had 2 consecutive estimate glomerular filtration rate (eGFR) measurements ≤ 45 (mL/min/1.73 m^2^) at least 90 days apart, with a prior eGFR ≥ 60. eGFR was estimated from serum creatinine using the 2009 CKD EPI (Chronic Kidney Disease Epidemiology Collaboration) equation.^13^ The second eGFR ≤ 45 (mL/min/1.73 m^2^) was defined as the index date. Patients requiring kidney replacement therapy with dialysis or kidney transplant were excluded. Prevalent heart failure was identified as having at least 1 inpatient or at least 3 separate outpatient visits using the International Classification of Diseases Ninth and Tenth Revisions diagnosis codes for heart failure in the preceding 5 years of incident CKD. Heart failure was further stratified using echocardiogram data (when available) as ejection fraction > 40% (HFpEF), ejection fraction ≤ 40% (HFrEF), or unknown if ejection fraction data was unavailable (Fig 1).

### Outcomes and Covariates

The primary outcomes were all-cause and cardiovascular-related mortality within 1 year of CKD identification. Mortality was identified from the KPSC mortality database, which combines information from California State Death Master Files, California State Multiple Cause of Death Master Files, Social Security Administration Death Master Files, KPSC Hospital and Emergency Room records, KPSC Membership System, Perinatal Data Mart, and Outside Claims Processing System. The cause of mortality was primarily obtained by linkage to the California State Multiple Cause of Death Master Files using *International Classification of Diseases, Tenth Revision* codes.^1^

Members’ demographic information such as age, sex, and race/ethnicity were identified from KPSC membership files. Preselected comorbid conditions such as hypertension, diabetes, coronary artery disease, atrial fibrillation, and weighted Charlson comorbidity score were determined by *International Classification of Diseases Ninth and Tenth Revisions* codes using electronic health record utilization database.^14^

### Statistical Analysis

Patients’ characteristics were compared among CKD patients without heart failure and CKD with heart failure further categorized as HFrEF, HFpEF, or unknown ejection fraction. Age- and sex-adjusted mortality rates were calculated by direct standardization using United States 2010 Census data. Hazard ratios (HRs) and their 95% confidence intervals (95% CIs) for 1-year all-cause mortality were estimated using a Cox proportional-hazards model adjusting for preselected confounders including age, sex, race/ethnicity, hypertension, diabetes, CKD stage, coronary artery disease, atrial fibrillation, and weighted Charlson comorbidity score at baseline. The subdistribution HRs of cardiovascular-related mortality within 1 year was estimated using the Fine-Gray subdistribution hazard model after adjustment for the same set of confounders, accounting for all other-cause mortality as a competing risk.

## Results

A total of 76,688 patients (52.0% non-Hispanic White, 23.4% Hispanic, 13.3% Black, and 8.8% Asian) were identified with incident CKD, of which 14,249 (18.6%) had a diagnosis of heart failure (Table 1). Among CKD patients with heart failure, 8,436 (59.2%) were HFpEF, whereas 3,328 (23.3%) were HFrEF (Fig 1). Compared with patients without heart failure, those with heart failure had higher Charlson comorbidity scores, co-existence of diabetes, hypertension, atrial fibrillation, and other cardiovascular diseases. There were a higher proportion of males and Black patients among CKD patients with heart failure. Mean systolic blood pressure was lower among the heart failure population and lowest among HFrEF patients (Table 1).

**Table 1:** Baseline Characteristics of Incident Chronic Kidney Disease Patients Based on Heart Failure Status (2007-2017)

	Heart Failure			No HF	Total
	No EF	EF > 40%	EF ≤ 40%		
N	2,485	8,436	3,328	62,439	76,688
Age at index date, mean (SD)	75.9 (11.04)	75.6 (11.31)	73.9 (11.22)	71.7 (12.36)	72.4 (12.24)
Sex, n (%)					
Male	1,242 (50.0%)	3,964 (47.0%)	2,166 (65.1%)	27,688 (44.3%)	35,060 (45.7%)
Female	1,243 (50.0%)	4,472 (53.0%)	1,162 (34.9%)	34,751 (55.7%)	41,628 (54.3%)
Race/ethnicity, n (%)					
White	1,524 (61.3%)	4,751 (56.3%)	1,687 (50.7%)	31,879 (51.1%)	39,841 (52.0%)
Asian	139 (5.6%)	576 (6.8%)	235 (7.1%)	5,832 (9.3%)	6,782 (8.8%)
Black	289 (11.6%)	1,204 (14.3%)	610 (18.3%)	8,116 (13.0%)	10,219 (13.3%)
Hispanic	411 (16.5%)	1,783 (21.1%)	736 (22.1%)	15,016 (24.0%)	17,946 (23.4%)
Other/unknown	122 (4.9%)	122 (1.5%)	60 (1.8%)	1,596 (2.6%)	1,900 (2.5%)
BMI, kg/m^2^					
Mean (SD)	30.1 (7.72)	30.3 (7.68)	28.1 (6.28)	29.2 (6.66)	29.3 (6.81)
< 18.5	26 (1.1%)	147 (1.7%)	57 (1.7%)	900 (1.4%)	1,130 (1.5%)
18.5-24.9	463 (18.6%)	1,723 (20.4%)	942 (28.3%)	13,111 (21.0%)	16,239 (21.2%)
25-29.9	618 (24.9%)	2,208 (26.2%)	982 (29.5%)	18,061 (28.9%)	21,869 (28.5%)
30-34.9	382 (15.4%)	1,518 (18.0%)	528 (15.9%)	11,373 (18.2%)	13,801 (18.0%)
35-39.9	210 (8.5%)	891 (10.6%)	236 (7.1%)	5,289 (8.5%)	6,626 (8.6%)
40+	193 (7.8%)	839 (10.0%)	143 (4.3%)	3,599 (5.8%)	4,774 (6.2%)
Unknown	593 (23.9%)	1,110 (13.2%)	440 (13.2%)	10,106 (16.2%)	12,249 (16.0%)
Average DBP within 1 y before index date					
Mean (SD)	65.2 (9.44)	64.8 (9.41)	64.5 (9.76)	68.6 (9.44)	67.9 (9.56)
< 60	524 (21.1%)	2,218 (26.3%)	963 (28.9%)	8,474 (13.6%)	12,179 (15.9%)
60 to < 80	1,306 (52.6%)	4,779 (56.7%)	1,764 (53.0%)	38,829 (62.2%)	46,678 (60.9%)
80+	129 (5.2%)	472 (5.6%)	208 (6.3%)	6,441 (10.3%)	7,250 (9.5%)
Unknown	526 (21.2%)	967 (11.5%)	393 (11.8%)	8,695 (13.9%)	10,581 (13.8%)
Average SBP within 1 y before index date					
Mean (SD)	126.1 (15.85)	1,26.9 (14.71)	118.0 (15.66)	129.5 (13.39)	128.6 (13.96)
< 120	659 (26.5%)	2,278 (27.0%)	1,658 (49.8%)	11,052 (17.7%)	15,647 (20.4%)
120 to < 140	959 (38.6%)	3,926 (46.5%)	1,019 (30.6%)	32,593 (52.2%)	38,497 (50.2%)
140+	341 (13.7%)	1,265 (15.0%)	258 (7.8%)	10,099 (16.2%)	11,963 (15.6%)
Unknown	526 (21.2%)	967 (11.5%)	393 (11.8%)	8,695 (13.9%)	10,581 (13.8%)
CKD stage/eGFR (mL/min/1.73 m^2^)					
<15	10 (0.4%)	85 (1.0%)	30 (0.9%)	738 (1.2%)	863 (1.1%)
15-29	252 (10.1%)	850 (10.1%)	311 (9.3%)	5,097 (8.2%)	6,510 (8.5%)
30-45	2,223 (89.5%)	7,501 (88.9%)	2,987 (89.75%)	56,604 (90.7%)	69,315 (90.4%)
Weighted Charlson comorbidity, median (IQR)	5.0 (4.0, 7.0)	6.0 (4.0, 7.0)	6.0 (5.0, 7.0)	4.0 (2.0, 5.0)	4.0 (2.0, 5.0)
Elixhauser comorbidity, median (IQR)	7.0 (6.0, 9.0)	9.0 (7.0, 11.0)	9.0 (7.0, 11.0)	5.0 (4.0, 7.0)	5.0 (4.0, 8.0)
Pre-existing comorbid conditions, n (%)					
Diabetes	1,320 (53.1%)	4,691 (55.6%)	1,857 (55.8%)	27,510 (44.1%)	35,378 (46.1%)
Hypertension	2,110 (84.9%)	7,743 (91.8%)	2,992 (89.9%)	47,725 (76.4%)	60,570 (79.0%)
Atrial fibrillation	583 (23.5%)	3,035 (36.0%)	1,245 (37.4%)	3,511 (5.6%)	8,374 (10.9%)
Coronary artery disease	1,252 (50.4%)	3,875 (45.9%)	2,066 (62.1%)	10,022 (16.1%)	17,215 (22.4%)
Malignancy	281 (11.3%)	1048 (12.4%)	418 (12.6%)	7524 (12.1%)	9271 (12.1%)

Abbreviations: BMI, body mass index; CKD, chronic kidney disease; DBP, diastolic blood pressure; EF, ejection fraction; eGFR, estimated glomerular filtration rate; body mass index; HF, heart failure; IQR, interquartile range; SBP, systolic blood pressure; SD, standard deviation.

One-year all-cause mortality occurred in 17.8% of CKD patients with heart failure compared with 6.3% of CKD patients without heart failure (Table 2). The age, sex-adjusted all-cause mortality rate (events per 1,000 person-years) in CKD patients with heart failure was 125.1 (95% CI, 100.1-150.2), compared with 35.9 (95% CI, 32.4-39.4) in those without heart failure. The highest age and sex-adjusted all-cause mortality rate per 1,000 person-years within 1 year was found among patients with HFrEF at 137.0 (95% CI, 92.7-181.2), followed by the patients with HFpEF, whose adjusted rate was 125.2 (95% CI, 90.7-159.8).

**Table 2:** One Year All-Cause and Cardiovascular-Related Mortality Incidence Rate

	One Year All-Cause and Cardiovascular-Related Mortality by Heart Failure and Chronic Kidney Disease Status				
			Outcome	Crude Incidence Rate^a^ (95% CI)	Adjusted Incidence Rate^a^^,^^b^ (95% CI)
All-cause death	No heart failure (N=62,439)		3,928 (6.3%)	66.7 (64.6, 68.8)	35.9 (32.4, 39.4)
	Heart failure (N=14,249)	Total	2,534 (17.8%)	201.9 (194.0, 209.8)	125.1 (100.1, 150.2)
		No EF (N=2,485)	335 (13.5%)	148.6 (132.7, 164.5)	116.8 (49.9, 183.7)
		EF > 40% (N=8,436)	1,414 (16.8%)	189.5 (179.6, 199.4)	125.2 (90.7, 159.8)
		EF ≤ 40% (N=3,328)	774 (23.3%)	278.3 (258.7, 298.0)	137.0 (92.7, 181.2)
Cardiovascular-related death	No heart failure (N=62,439)		572 (0.9%)	9.7 (8.9, 10.5)	4.4 (3.3, 5.5)
	Heart failure (N=14,249)	Total	1,449 (10.2%)	116.0 (110.0, 121.9)	52.4 (40.5,64.3)
		No EF (N=2,485)	160 (6.4%)	71.0 (60.0, 82.0)	54.5 (8.2, 100.8)
		EF > 40% (N=8,436)	754 (8.9%)	101.1 (93.8, 108.3)	47.1 (30.1, 64.1)
		EF ≤ 40% (N=3,328)	535 (16.1%)	192.4 (176.1, 208.7)	72.3 (57.3, 87.4)

Abbreviations: CI, confidence interval; EF, ejection fraction.

a Rates are per 1,000 person-years

b Age, sex-adjusted using US 2010 Census Data

One-year cardiovascular-related mortality was 10.2% in CKD patients with heart failure compared with 0.9% in CKD patients without heart failure. The age and sex-adjusted incidence rate of cardiovascular-related mortality was 11 times higher among CKD patients with heart failure than CKD patients without heart failure, 52.4 (95% CI, 40.5-64.3) versus 4.4 (95% CI, 3.3-5.5) per 1,000 person-years, respectively. The incidence of cardiovascular-related mortality was highest among CKD patients with HFrEF.

Compared with CKD patients without heart failure, the HR of 1-year all-cause mortality was 1.70 (95% CI, 1.60-1.80) for CKD patients with heart failure in the multivariable model (Table 3). Further analysis by ejection fraction demonstrated that the 1-year all-cause mortality HR was 2.43 (95% CI, 2.23-2.65) for HFrEF and 1.59 (95% CI, 1.48-1.70) for HFpEF, compared with CKD patients without heart failure. The 1-year cardiovascular mortality HR was 6.69 (95% CI, 5.93-7.54) for CKD patients with heart failure compared with CKD patients without heart failure after considering other causes of mortality as a competing risk. Compared with CKD patients without heart failure, the 1-year cardiovascular mortality HR was 11.47 (95% CI, 9.90-13.28) and 6.10 (95% CI, 5.36-6.94) for CKD patients with HFrEF and HFpEF, respectively.

**Table 3:** One Year All-Cause and Cardiovascular-Related Mortality Hazard Ratio

	1 year All-Cause and Cardiovascular-Related Mortality by Heart Failure and Chronic Kidney Disease Status					
			Outcome		HR^a^ (95% CI)	Adjusted HR^b^ (95% CI)
All-cause death rowhead	No heart failure (N=62,439)		3,928 (6.3%)		Ref	Ref
	Heart failure (N=14,249)	Total	2,534 (17.8%)		3.00 (2.86, 3.16)	1.70 (1.60, 1.80)
		No EF (N=2,485)	335 (13.5%)		2.22 (1.98, 2.48)	1.39 (1.24, 1.56)
		EF > 40% (N=8,436)	1,414 (16.8%)		2.82 (2.66, 3.00)	1.59 (1.48, 1.70)
		EF ≤ 40% (N=3,328)	774 (23.3%)		4.12 (3.81, 4.45)	2.43 (2.23, 2.65)

			Outcome	HR^c^ (95% CI)	Adjusted HR^d^ (95% CI)
Cardiovascular-related death	No heart failure (N=62,439)		572 (0.9%)	Ref	Ref
	Heart failure (N=14,249)	Total	1,449 (10.2%)	11.66 (10.59, 12.85)	6.69 (5.93, 7.54)
		No EF (N=2,485)	160 (6.4%)	7.21 (6.05, 8.59)	4.46 (3.70, 5.38)
		EF > 40% (N=8,436)	754 (8.9%)	10.17 (9.13, 11.34)	6.10 (5.36, 6.94)
		EF ≤ 40% (N=3,328)	535 (16.1%)	19.21 (17.07, 21.61)	11.47 (9.90, 13.28)

Abbreviations: CI, confidence interval; CKD, chronic kidney disease; EF, ejection fraction, HR, hazard ratio; Ref, reference.

a Unadjusted Cox proportional-hazards model

b Cox proportional-hazards model adjusted for age, sex, race/ethnicity, diabetes, hypertension, atrial fibrillation, coronary artery disease, CKD stage, and weighted Charlson comorbidity score.

c Unadjusted Fine-Gray subdistribution hazard model

d Fine-Gray subdistribution hazard model adjusted for age, sex, race/ethnicity, diabetes, hypertension, atrial fibrillation, coronary artery disease, CKD stage, and weighted Charlson comorbidity score.

## Discussion

Our study evaluated patients with incident advanced CKD (eGFR < 45 mL/min/1.73 m^2^) and found that 18.6% had prevalent heart failure. In addition, we found that over 70% of patients with CKD with heart failure and a known ejection fraction had preserved ejection fraction compared to the general population, in which about half of diagnosed heart failure patients described have reduced ejection fraction.^15^^,^^16^ Our findings were similar to the Chronic Renal Insufficiency Cohort, which demonstrated considerably higher incident HFpEF rates (8.6) compared with HFrEF (6.7 per 1000 person-years) among the CKD population.^17^

Our study using real-world data from a large integrated health system highlights the vulnerability of patients with both CKD and heart failure. Our study demonstrates that CKD patients with heart failure have a substantially higher risk of both short-term, all-cause and cardiovascular-related mortality compared with patients with CKD without heart failure. Among patients with CKD with HFrEF, there was a greater than 2-fold risk of 1-year all-cause mortality and up to 11-fold increased risk of cardiovascular-related mortality compared with patients with CKD without heart failure. The high rate of cardiovascular-related mortality likely reflects progressive heart failure or sudden cardiac death, which is not uncommon among the CKD population.^18^, ^19^, ^20^ Furthermore, our patients with CKD without heart failure may represent a much different subgroup given the commonality of heart failure among the CKD population. Among our study population of patients with CKD without heart failure, the cardiovascular mortality accounted for only 12.7% of all-cause mortality, which is lower than reported (up to 47%) among all patients with CKD, including those with kidney failure.^1^^,^^3^^,^^4^

The ideal management for CKD patients with heart failure is largely unknown. Current therapy recommendations for patients with heart failure are derived from evidence-based cardiovascular outcome trials, termed “guideline-directed medical therapy.” They were introduced in 2013 to optimize medical therapy for patients with heart failure.^6^ In addition, the optimal management of patients with HFpEF is uncertain. Overall, patients with CKD have been mostly excluded from trials that lead to guideline-directed medical therapy recommendations, with only a few trials demonstrating evidence of benefit for guideline-directed medical therapy medications in patients with HFrEF and CKD.^21^

There are potential limitations of our study that may confound the interpretation of our findings. First, this was a retrospective cohort study in a population with prevalent heart failure at CKD incidence. The heart failure may have caused the incident CKD in these patients (cardiorenal syndrome) and thus, that in and of itself may have accounted for the substantially higher risk of cardiovascular-related deaths given the short-term follow-up of our study. In addition, because we used the intent-to-treat method only using baseline information, the time-varying variables such as medication adherence and particularly guideline-directed medical therapy for the heart failure population during follow-up were not measured. However, because the follow-up was relatively short at 1 year, we expected the change to be small. We did not have comprehensive information on albuminuria and did not account for it in the outcomes analyses though albuminuria is a well described risk factor for cardiovascular and mortality outcomes. Our study population only included an insured population within Southern California and may not reflect the general CKD and heart failure populations in the community.

Despite these potential limitations, our study findings are derived from a large, diverse population of patients with CKD. The integrated health system and insured population minimizes many of the issues related to access to care and inconsistencies in health delivery. In addition, KPSC has standardized approaches to many chronic conditions such as hypertension and CKD, which can minimize heterogeneity of practice patterns among providers.^22^^,^^23^ We identified our incident CKD population using a comprehensive approach based on serial eGFR measurements over specified time periods. We used an electronic health record-based approach to identify heart failure but also had echocardiogram data on over 80% of the heart failure patients. Therefore, the findings from our diverse population of a real-world clinical practice environment may have greater translational value than more artificial environments (eg, clinical trials) in terms of reflecting what occurs in the community.

Among a large diverse real-world population of patients with incident CKD, we observed a high rate of prevalent heart failure. HFpEF was the predominant form of heart failure over HFrEF among CKD patients. One-year all-cause mortality and cardiovascular-related mortality risk was highest among HFrEF followed by HFpEF compared with CKD patients without heart failure. Given the advancements in therapies for heart failure such as neprilysin inhibitors, sodium/glucose cotransporter 2 inhibitors, and newer generation mineralocorticoid receptor antagonists, future studies are needed to assess the efficacy of these drugs in real-world populations. We hope that such future work will provide more insights into the evolving care for heart failure and CKD,^24^ ultimately to help better understand and manage this vulnerable population.
