# Supplementary material for: All-Cause and Cardiovascular-Related Mortality in CKD Patients With and Without Heart Failure: A Population-Based Cohort Study in Kaiser Permanente Southern California
Source: Kidney Med. 2023 Mar 9;5(5):100624. doi: 10.1016/j.xkme.2023.100624 (PMC10151415; doi:10.1016/j.xkme.2023.100624)
Supplement: Supplementary File (PDF) — Table S1. [file mmc1.pdf]

Table S1. Diagnosis and procedure codes associated with interested comorbidities

**Comorbidity**	**Diagnosis/Procedure Codes**
Diabetes	**≥1 inpatient or ≥ 2 outpatient diagnosis**<br>ICD-9: 250.X, 357.2X, 362.0X, 366.41X, 648.0X<br>ICD-10: E10.X, E11.X, E13.X, O24.319X, O24.912X, O24.913X, O24.92X, O24.93X
Hypertension	**≥1 inpatient or ≥ 2 outpatient diagnosis**<br>ICD-9: 250.41, 250.40, 402.00, 402.01, 402.1, 402.11, 402.90, 402.91, 403.00, 403.01, 403.10, 403.11, 403.90, 403.91, 404.00, 404.01, 404.02, 404.11, 404.12, 404.13, 404.90, 404.91, 404.92, 404.93, 405.01, 405.09, 405.10, 405.19, 405.91, 405.99<br>ICD-10: E10.22, E11.22, H35.031, H35.032, H35.033, H35.039, I10, I11.0, I11.9, I12.0, I12.9, I13.0, I13.10, I13.11, I13.2, I15.0, I15.1, I15.2, I15.8, I15.9, I16.0, I16.1, I16.9, N26.2
Atrial Fibrillation	**≥1 inpatient or ≥ 2 outpatient diagnosis**<br>ICD-9: 427.3, 427.31, 427.32<br>ICD-10: I48.0 I48.1, I48.2, I48.3, I48.4, I48.91, I48.92
Coronary Artery Disease	**Myocardial infarction: ≥1 inpatient principal discharge diagnosis**<br>ICD-9: 410.X<br>ICD-10: I21.X, I22.X<br><br>**Ischemic heart disease: ≥1 inpatient principal discharge or ≥ 2 outpatient diagnosis**<br>ICD-9: 411.X, 412.X, 413.X, 414.X<br>ICD-10: I20.X, I24.X, I25.X<br><br>**PCI: ≥1 inpatient or ≥ 2 outpatient procedure**<br>ICD-9: 00.66<br>ICD-10: 02710, 02720, 02730, 02723, 02724, 02733, 02734, 02C03, 02C04, 02C13, 02C14, 02C23, 02C24, 02C33, 02C34, 021K4, 021L4, 02QA4, 02QB4, 02QC4, 3E073<br>CPT: 92920, 92924, 92928, 92933, 92937, 92941, 92943, 92980, 92982, 92995, 92984, 92996<br>HCPCS: C9600, C9602, C9604, C9606, C9607<br><br>**CABG: ≥1 inpatient procedure**<br>ICD-9: 36.03, 36.1, 36.2, 36.3<br>ICD-10: 02100, 02110, 02130, 2103, 02104, 02113, 02114, 02123, 02124, 02133, 02134, 02C00, 02C10, 02C20, 02C30, 021K0, 021L0, 03E070<br>CPT: 33510, 33511, 33512, 33513, 33514, 33516, 33517, 33518, 33519, 33521, 33522, 33523, 33530, 33533, 33534, 33535, 33536
Malignancy	Cancer and metastatic cancer from Charlson comorbidity
